# Challenges Associated With Ellipsoid Zone Intensity Measurements Using Optical Coherence Tomography

**DOI:** 10.1167/tvst.10.12.27

**Published:** 2021-10-19

**Authors:** Karen E. Lee, Heather Heitkotter, Joseph Carroll

**Affiliations:** 1 Medical College of Wisconsin, Milwaukee, WI, USA; 2 Cell Biology, Neurobiology & Anatomy, Medical College of Wisconsin, Milwaukee, WI, USA; 3 Ophthalmology & Visual Sciences, Medical College of Wisconsin, Milwaukee, WI, USA

**Keywords:** optical coherence tomography, ellipsoid zone, biomarker

## Abstract

Optical coherence tomography (OCT) allows noninvasive visualization of individual retinal layers and has become a mainstay in the diagnosis and management of a wide range of retinal and systemic diseases. As the number of available treatments increases, there is growing interest in developing sensitive OCT-based biomarkers for assessing therapeutic response. In particular, the hyperreflective outer retinal band just posterior to the external limiting membrane, also known as the ellipsoid zone (EZ), is a widely used biomarker of photoreceptor structure. The integrity of the EZ, EZ lesion size, and width/area of retained EZ are established metrics that have been correlated with visual acuity and other aspects of retinal function (e.g., microperimetry and electroretinography). More recently, EZ reflectivity has emerged as a potentially more sensitive biomarker of photoreceptor structure, as reflectivity has been shown to undergo changes in retinal degenerative conditions prior to more marked changes in EZ integrity. However, multiple challenges exist that prohibit widespread clinical utilization. Interdevice variability can impact OCT image appearance due to differences in hardware, acquisition parameters, and image processing methods. In addition, image analysis practices vary widely across studies—this lack of standardization prevents robust comparison of results between studies and inhibits more widespread adoption of extracted biomarkers. Finally, there is ambiguity as to how well EZ intensity/reflectivity correlates with underlying photoreceptor structure as assessed with adaptive optics-based imaging methods. Here we review these challenges and discuss their impact on the use of EZ reflectivity measurements.

**Translational Relevance:**

Qualitative evaluation of the ellipsoid zone band on optical coherence tomography is a valuable clinical tool for assessing photoreceptor structure, though more quantitative metrics are emerging. Awareness of the challenges involved in interpreting quantitative metrics is important for their clinical translation.

## Introduction

**Figure 1.**
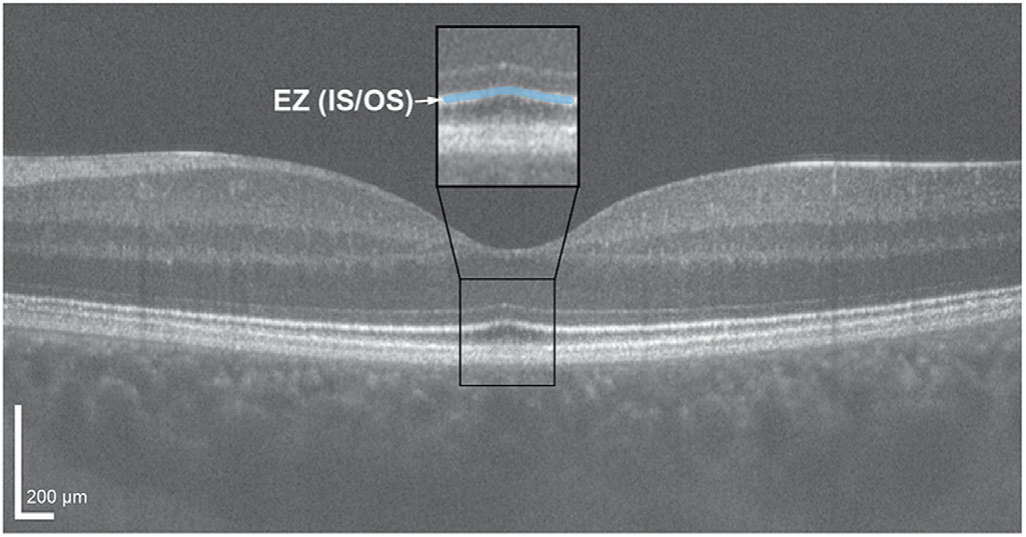
Horizontal line scan through the foveal center of the left eye of a 24-year-old female with normal vision acquired using a Bioptigen SD-OCT device. Scan was acquired using a setting of 1000 A-scans/B-scan, and the image is a registered average of 20 B-scans. OCT line scans enable delineation of the various retinal layers, including the four hyperreflective outer retinal bands. Of particular interest is the second hyperreflective band, also known as the ellipsoid zone (EZ) or inner segment/outer segment (IS/OS) junction (*highlighted blue* in the *inset black box*). *Scale bar* = 200 µm.

Optical coherence tomography (OCT) enables volumetric visualization of the retina in vivo, with commercial clinical systems having an axial resolution of better than 5 µm.^1^^,^^2^ The ability to resolve individual retinal layers allows quantitative monitoring of a number of retinal and systemic diseases, which facilitates clinical diagnosis and treatment. This is perhaps most evident in congenital and acquired conditions affecting photoreceptor structure, especially as more treatment options emerge. One of the most commonly used biomarkers to quantify remnant photoreceptor structure is outer nuclear layer (ONL) thickness,^3^^,^^4^ though resolution of the Henle fiber layer is required for accurate measurements.^4^ Loss of photoreceptor nuclei manifests as thinning of the ONL, though this tends to occur late in the degenerative process,^5^ making it a poor biomarker for early detection of disease. Furthermore, disambiguating rod versus cone contributions to ONL thickness is not currently possible using OCT. Limitations such as these have led to a growing interest in assessing other aspects of photoreceptor anatomy with OCT. In particular, the hyperreflective outer retinal band just posterior to the external limiting membrane (ELM) has become an accepted biomarker of photoreceptor structure (Fig. 1). There is some controversy with respect to the name and anatomical origin of this hyperreflective band—some suggest it originates from the ellipsoid zone (EZ) of the photoreceptor while others suggest it corresponds to the junction between the photoreceptor inner segment (IS) and outer segment (OS; also known as IS/OS).^6^^–^^9^ Regardless of the exact subcellular origin, changes in the appearance of this band (which we will refer to as the EZ) on OCT are often used as an indicator of photoreceptor pathology and thus may serve as a means to monitor disease progression or therapeutic response. The purpose of this perspective is to review some of the main technical considerations that impact widespread reliable utilization of EZ metrics.

## Imaging Methods

To demonstrate some of the concepts in this review, we utilized retinal images obtained from human subjects. Demographic details of each subject and imaging devices used are provided within their respective figure caption. Images were collected under studies which conformed to the tenets of the Declaration of Helsinki and were reviewed and approved by the Institutional Review Board at the Medical College of Wisconsin. Written informed consent was obtained from all subjects prior to their participation in those imaging studies. The images from those original studies reside in an IRB-approved bank and were extracted for use in this review under an IRB-approved bank access protocol (PRO030741).

## Current EZ Metrics

**Figure 2.**
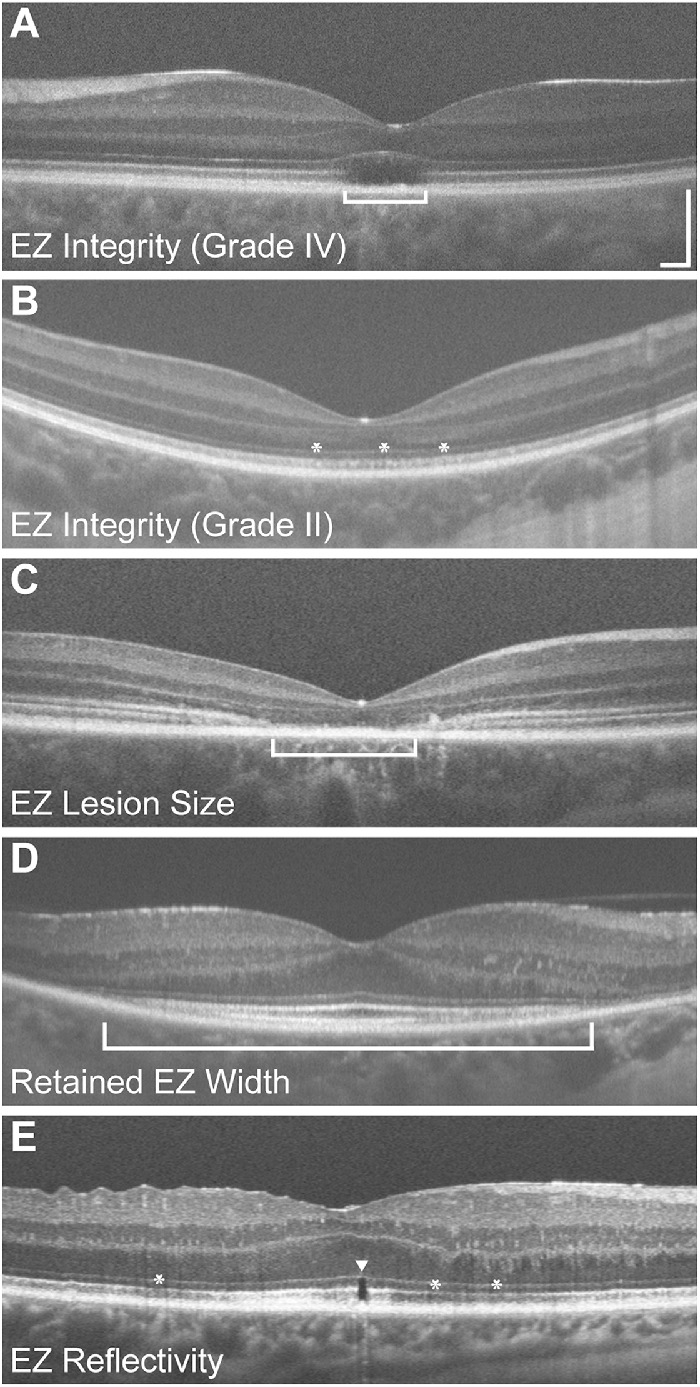
Examples of various EZ metrics: foveal EZ integrity (**A**, **B**), EZ lesion size (**C**), retained EZ width (**D**), and EZ reflectivity (**E**). All OCTs are horizontal line scans collected on a Bioptigen SD-OCT device using a setting of 1000 A-scans/B-scan, the *scale bar* is 200 µm and applies to all images. Panel (A) shows the left eye of a 24-year-old female with congenital achromatopsia (due to compound heterozygous mutations in *CNGA3*, p.Val451Gly and p.Arg427Cys). The absence of the EZ within the bracket represents a grade IV EZ (a.k.a., hyporeflective zone) using the Sundaram et al. grading scheme.^21^ Panel (**B**) shows the right eye of a 6-year-old male with blue cone monochromacy (caused by a deletion of the locus control region upstream of the *OPN1LW* / *OPN1MW* gene array). The mottled appearance of the EZ (*asterisks*) would be consistent with a grade II on the Sundaram et al. scheme. Panel (**C**) shows the right eye of a 15-year-old female with Stargardt disease (caused by compound heterozygous mutations in *ABCA4*, p.Arg602Trp, and p.Gly863Ala). The EZ lesion extends across the *width of the bracket*. Panel (**D**) is from the right eye of a 51-year-old female with autosomal dominant retinitis pigmentosa (due to the p.Pro23His mutation in *RHO*), and the extent of the retained EZ width is marked by the *bracket below* the RPE. Panel (**E**) is from the right eye of a 63-year-old male five months post recovery from retinal detachment repair. There is a small (<50 µm) EZ disruptions at the fovea (marked by the *arrowhead*), with diffuse attenuation of EZ reflectivity both nasal and temporal to the fovea (*asterisks*). The OCT images are registered averages of varying numbers of B-scans (14 for panel A, 10 for panel B, 15 for panel C, 21 for panel D, and 40 for panel E).

Common metrics for evaluating the EZ include band integrity, EZ lesion area, and width/area of retained EZ (see Fig. 2). One of the more basic measures of EZ integrity is a subjective assessment of whether the band is intact, disrupted, or absent.^10^^–^^12^ Longitudinal reflectivity profiles (LRPs), which evaluate the gray value intensity axially through the OCT image,^13^ can be used to facilitate this assessment, though this is really only practical for focal assessment of EZ integrity.^14^^,^^15^ Categorical grading schemes capture regional properties of the EZ and have been developed to describe EZ band disruption at the fovea in certain retinal conditions such as diabetic macular edema,^16^ retinitis pigmentosa (RP),^17^ and epiretinal membrane.^11^ In multiple studies EZ integrity is categorically graded (present, absent, attenuated) to correlate with visual acuity, either related to disease severity or recovery posttreatment.^11^^,^^16^^,^^18^^–^^20^ Another grading scheme has been developed specifically for patients with achromatopsia, where grade 1 indicates an intact foveal EZ, grade 2 shows a small focal disruption or mottled appearance, grade 3 indicates absence of the EZ with a collapsed ELM and normal retinal pigment epithelium (RPE) appearance, grade 4 denotes a hyporeflective zone or foveal cavitation, while grade 5 indicates an absence of the EZ with complete macular atrophy.^21^ Regardless of the method used to assess EZ integrity, there have been numerous studies across a wide range of diseases examining how EZ integrity correlates to measures of visual function, either to better understand disease pathophysiology or to develop a prognostic indicator of functional outcomes.^16^^,^^18^^,^^20^^,^^22^^–^^28^

EZ lesion size is a quantitative metric defined as the extent of EZ absence/disruption and is commonly reported as total lesion area (px^2^ or mm^2^). Typically, EZ lesion size is used in populations where breaks in EZ reflectance occur near the fovea, while the peripheral EZ remains intact. EZ lesion size has been correlated with disease severity and progression, with previous studies demonstrating that EZ lesions show associated loss of retinal function and decreased visual acuity in patients with Best vitelliform macular dystrophy,^29^ solar maculopathies,^30^ macular telangiectasia (MacTel) type 2,^31^ and retinal vein occlusion.^32^ En face OCT has been used to cross-sectionally quantify the attenuation of macular EZ lesion area in Stargardt disease,^33^ and the rate of EZ loss exhibited high intra- and intergrader reliability,^34^ suggesting its potential use as a valuable structural outcome measure in clinical trials.

Conversely, retained EZ width or retained EZ area is used to characterize the central region of preserved EZ. In conditions such as RP, the rate of decline in EZ width correlates with the rate of change for the equivalent area of viable retina,^35^ and has been used as a surrogate for deterioration of the visual field.^36^ Excellent repeatability and reproducibility of EZ width measurements have been demonstrated,^37^ supporting its use as a reliable metric to monitor disease progression over time in clinical trials of RP. Despite these strengths, EZ width is measured on a single OCT line scan, which samples only a small portion of the region of preserved photoreceptors and may not capture nonuniformities in the pattern of EZ constriction. On the other hand, measuring the area of preserved EZ with volumetric OCT scans provides a more complete assessment of the retained EZ structure. Sampling the entire EZ rather than a single B-scan can reduce the risk of error, and may more accurately reflect the extent of a functional visual field.^38^ Consequently, preserved EZ area has been suggested as a potential anatomic outcome measure for choroideremia^39^ and RP^38^ clinical trials—as a slower rate of change in EZ retained area could indicate positive treatment response.^38^

Despite their widespread use, the above EZ metrics have some important limitations. For EZ integrity, many categorical grading schemes are subjective, which can result in ambiguity between graders when assessing characteristics of the EZ on OCT scans.^40^ Such ambiguity can limit comparison of data between studies. In addition, EZ integrity on its own cannot be used to discriminate between rod and cone photoreceptor structure.^41^ While quantitative, EZ width and area metrics (whether representing EZ lesion(s) or the retained EZ) require proper lateral scaling of the OCT image, which necessitates knowledge of the retinal magnification factor for a given eye. Retinal magnification varies between patients due primarily to differences in axial length,^42^ but it also can vary between devices due to differences in the optical design and optical model used to derive the nominal image scale.^43^ Proper scaling of OCT and OCT-angiography images is not widespread in the literature,^42^ which limits the ability to compare lateral measurements (such as EZ lesion size or retained EZ width/area) across studies. This may not affect longitudinal assessment of EZ structure on a patient level, assuming the patient's axial length remains constant. However, as trials for inherited retinal degenerations expand to pediatric populations, this will become a major limitation in monitoring disease progression and therapeutic response in individuals where the eye is still growing.

## EZ Reflectivity

While the above EZ metrics have been used extensively, there is growing interest in using EZ band intensity or reflectivity as a potentially more sensitive biomarker for evaluating photoreceptor structure, especially in early disease states or following surgical repair of macular hole and retinal detachment (see Fig. 2E).^20^ EZ reflectivity is affected by photoreceptor waveguiding and light scattering,^44^ and has been found to be maximally reflective when the OCT beam enters through the pupil center.^45^^–^^47^ Trauma resulting in commotio retinae often leads to a temporary increase in EZ reflectivity, or EZ disruption, that resolves over time.^48^^–^^52^ These changes to EZ reflectivity can be monitored, and has been suggested as a biomarker for tracking photoreceptor recovery following clinical intervention.^21^^,^^53^^,^^54^ Conditions with dysfunctional or reduced cones including age-related macular degeneration (AMD),^55^^,^^56^ RP,^57^ and achromatopsia^21^^,^^58^ often present with reduction in EZ reflectivity, supporting its use as a biomarker for photoreceptor structure and function. Furthermore, reduction in EZ reflectivity has been shown to occur prior to EZ dropout in conditions including nonneovascular AMD and epimacular membrane,^59^^–^^61^ suggesting that EZ reflectivity might provide a sensitive measure of subclinical deterioration of photoreceptors. Similarly, areas where EZ loss was initially observed in eyes with MacTel type 2 were found to later develop neovascular membranes—these areas must be treated before irreversible damage to photoreceptors occurs.^62^ Changes in EZ reflectivity have also been associated with measures of retinal function including visual acuity and retinal sensitivity in conditions such as MacTel type 2,^63^ early AMD,^64^ Best vitelliform macular dystrophy,^65^ and macular hole following surgical repair.^66^ Additionally, changes to EZ reflectivity have been correlated to retinal dysfunction and altered blood flow in type 1 diabetes without retinopathy.^67^ There are emerging techniques for detection of changes to EZ reflectivity across larger retina areas,^68^ including automated methods for quantifying EZ reflectivity.^69^^,^^70^ Taken together, these findings suggest that quantification of EZ reflectivity has clinical utility for disease detection (both earlier and more accurately) and tracking in a variety of retinal pathologies. Despite growing interest in the use of EZ reflectivity as a potential biomarker of photoreceptor function and structure, multiple challenges to its clinical adoption exist, which we review below.

## Challenge 1: Devices and Acquisition

There are several commercially available OCT systems^43^ that vary in specific light sources, acquisition speeds, B-scan averaging, and image postprocessing. Furthermore, some postprocessing steps are proprietary and opaque—thus the displayed image is not directly representative of raw data (at least from the end user's perspective). This confounds extraction of accurate reflectance values in many cases. While these factors may not impact studies within the same clinic over time, they can impact the ability to compare data across studies employing different devices. Indeed, differences in the retinal reflectance profile of OCT images across devices have been previously reported.^71^^,^^72^ Normalization of EZ reflectivity measurements may help compensate for some of these differences, which will be further discussed in Challenge 3.

**Figure 3.**
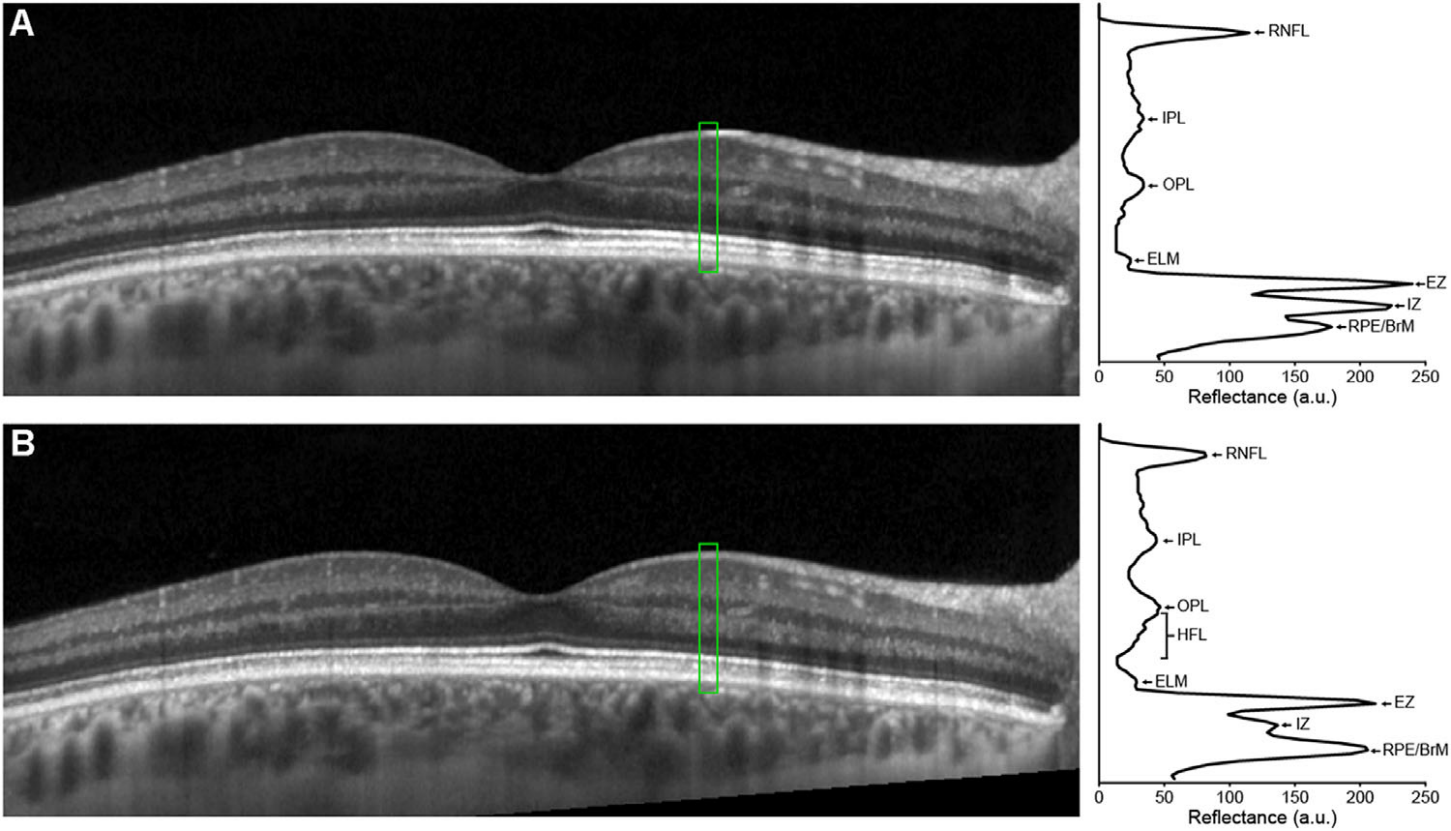
Horizontal line scan through the foveal center of the right eye of a 30-year-old female with normal vision acquired on a Spectralis OCT device collected with (**A**) the OCT beam entering the pupil centrally and (**B**) the OCT entrance beam displaced temporally (in follow-up mode). A horizontal line scan through the fovea was collected in EDI mode using the Spectralis ART feature to produce a final image with 100 frames averaged together. The follow-up scan is automatically aligned to the baseline image with the onboard Spectralis software—though the difference in pupil entry between scans can be detected by inspecting the angled cropping of the follow-up image (*bottom right corner of panel B*). To the right of each OCT scan is the longitudinal reflectivity profile (LRP) from the region *highlighted in green* (11 pixels wide). LRPs were generated with OCT Reflectivity Analytics (ORA) software.^130^ In both panels, the retinal nerve fiber layer (RNFL), inner plexiform layer (IPL), outer plexiform layer (OPL), external limiting membrane (ELM), EZ, interdigitation zone (IZ), and retinal pigment epithelium (RPE)/Bruch's membrane (BrM) are labeled. In panel B, the Henle fiber layer (HFL) is also labeled due to the increased reflectivity of the HFL with the eccentric pupil entry. The two LRPs demonstrate how layer reflectivity can vary due to pupil entry position, which could be misinterpreted as a change in retinal structure. Such variation is especially problematic for longitudinal studies that utilize automated alignment tools.

Pupil entry position is an important acquisition feature that is not regularly recorded during OCT acquisition and differs in user control across devices. Changes in pupil entry position of the OCT beam will result in an altered reflectivity profile of the retinal image (Fig. 3).^47^^,^^73^^,^^74^ Changes in retinal layer reflectivity depend on a number of factors including beam entry position, retinal layer composition, retinal pathology, and scan angle.^45^^,^^75^ Despite the well-known impact of pupil entry position on layer reflectivity, this information is not reliably captured by most devices, nor has it been controlled for in most previous studies examining EZ biomarkers. Without standardization of pupil entry point acquisition and understanding the relationship between entry point and reflectivity, there will be limitations on the reproducibility of EZ reflectivity measurements.

**Figure 4.**
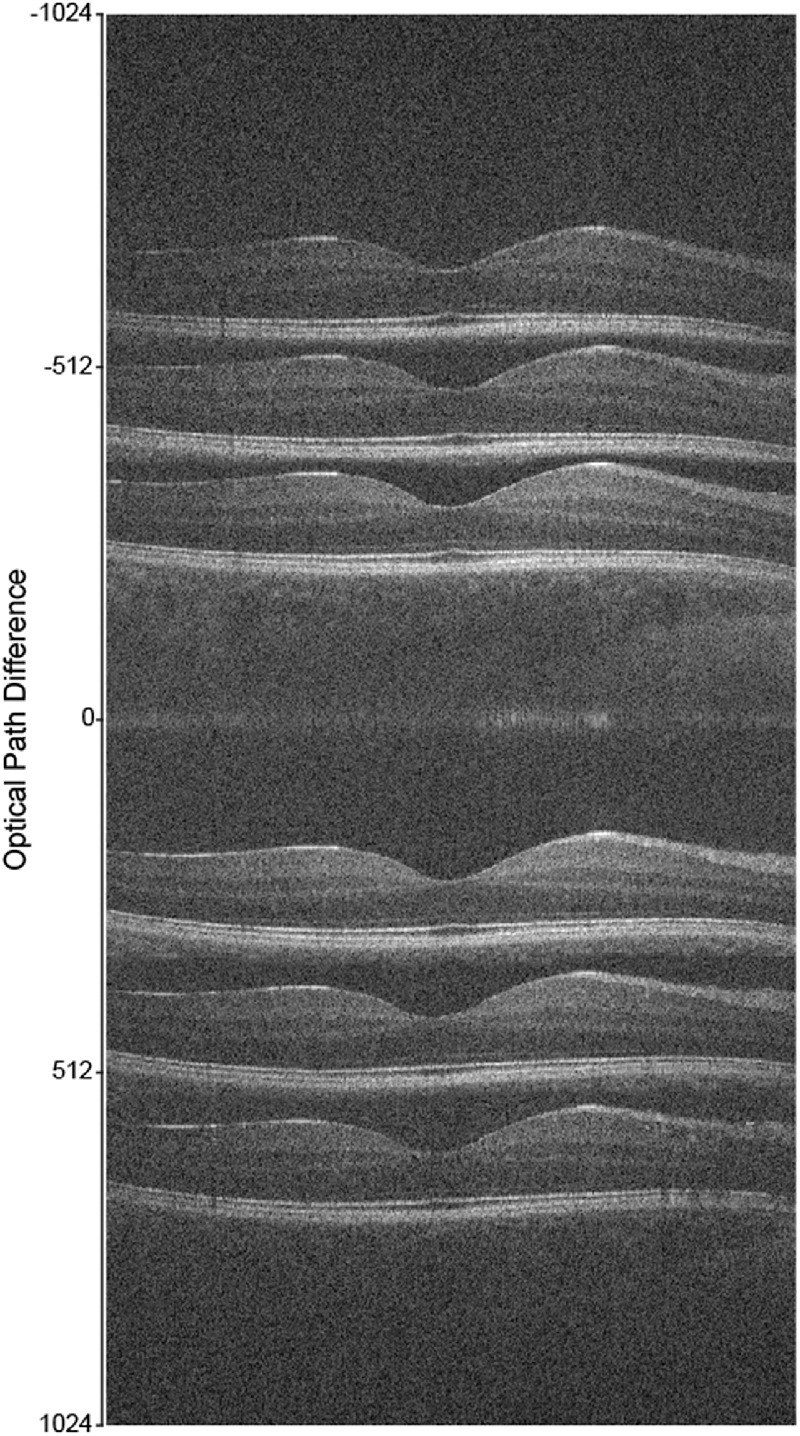
Horizontal line scans through the foveal center of the right eye of a 27-year-old female with normal vision acquired on a Bioptigen SD-OCT device in either standard or enhanced depth imaging (EDI) mode. By altering the axial distance of the device in reference to the retina, it is possible to vary the position of the retina in the scan window. Layer reflectivity will be highest when OCTs are collected nearest the zero-delay line (marked as 0 on the y axis). In non-EDI mode (images shown *below the zero-delay line*), reflectivity is maximal near the top of the scan window, where inner retinal layers will have greater intensity than the outer retina. In EDI mode (images shown *above the zero-delay line*), reflectivity is maximal near the bottom of the scan window and will show increased layer intensity in the outer retina and choroid. Each scan is a single B-scan (1000 A-scans/B-scan).

Like pupil entry point, enhanced depth imaging (EDI) is also an acquisition parameter that cannot be uniformly controlled. EDI is a feature available on most OCT devices and is used to improve the image quality of the deeper retinal structures including the choroid.^76^ Conventional SD-OCT imaging places the zero delay line close to the inner retinal layers, and is characterized by decreased sensitivity and resolution as the distance from the zero delay line increases. On the other hand, EDI works by using the inverted image and placing the choroid closer to the zero delay line, thus increasing resolution of the deeper retinal structures (Fig. 4).^76^ Even within the same acquisition mode (EDI or non-EDI), changes in the position of the OCT scan on the spectrometer also impact EZ reflectivity due to the roll-off in sensitivity as a function of spectrometer depth (Fig. 4). As such, it is critical to utilize the same acquisition mode (EDI or non-EDI) and control for spectrometer depth, especially if tracking EZ reflectivity over time in the same patient.

## Challenge 2: Logarithmic Versus Linear Display

OCT captures a large dynamic range of backscattered light to render an image, and these images are regularly presented in a logarithmic scale for easier perception of retinal layers compared to raw/linear data.^77^ While this transform enhances perception of contrast toward the lower end of the dynamic range, it results in misrepresentation of real differences in reflectivity and a loss of information.^69^^,^^77^ Furthermore, by distorting gray values, hyperreflective outer retinal bands are broadened and their vertical position can be altered within the scan.^8^^,^^21^^,^^77^ Measurements of EZ intensity made from logarithmic scale images should therefore be evaluated with caution.

Some studies have utilized linear data for EZ reflectivity analyses.^21^^,^^69^^,^^78^ One such study showed a reduction in EZ reflectivity in AMD subjects compared to controls and validated an automated method for extraction of EZ reflectivity to ultimately use on volumetric SD-OCT images.^69^ A study of Oguchi disease demonstrated that in light-adapted OCT images acquired with linear scale, the OS layer exhibits reduced Michelson contrast likely due to increased scattering of the EZ.^78^ It has also been shown that contrast-enhanced reflectivity obtained from logarithmic transformed images systemically overestimated band thicknesses and altered their position.^21^ Although this transformation can be mathematically converted into linear raw data using device specifications provided by the OCT manufacturer,^21^ the exact transform is not always disclosed, so even studies that attempt to convert their logarithmic images to a linear scale may be introducing additional errors in layer reflectivity.

## Challenge 3: Normalization Technique

As discussed above, interdevice variation in EZ intensity has been demonstrated. To correct for this, it has been shown that normalizing EZ reflectivity as a ratio of the intensity of the EZ band to a retinal layer that exhibits relative constancy through disease states is necessary.^71^ This normalization allows for comparison across subjects, devices, time points, and can also compensate for differences in spectrometer depth discussed above. However, this can be a complicating factor because each OCT device has proprietary methods of image acquisition and thus different ways to optimize parameters including working distance, reference arm, and spectrometer depth. This must be considered when comparing reflectivity measurements longitudinally, especially for disease states where pathological changes occur gradually over time.

Despite the need for EZ reflectivity normalization for data analysis, there is currently no consensus on a standardized method. For example, some studies have utilized the ELM,^56^^,^^64^^,^^69^^,^^79^ RPE,^1^ or a combination of the retinal nerve fiber layer (RNFL) and vitreous^80^—each with justification for the chosen layer for normalization. One group normalized to the local area around the specific EZ segment defined as extending 275 µm to either side of the segment and extending axially between the Bruch's membrane/choroid interface and posterior border of the RNFL.^58^ Similarly, another group normalized to the mean intensity of the whole retina at the same position for which EZ intensity measurements were taken.^57^ One study evaluating achromatopsia normalized to a local region of the retinal ganglion cell and inner plexiform layers.^21^ This study demonstrated a significant difference in mean EZ, but not ELM, intensity between achromatopsia subjects and controls, suggesting this normalization method was effective. Within-scan normalization has also been used,^81^ and while this is an effective way to address the issue of spectrometer depth and EDI/non-EDI mode, it would not correctly account for EZ reflectivity differences due to scan angle. Across these methods for normalizing EZ intensity, some may be better than others due to physiological differences in layer reflectivity. For example, the RNFL has the highest degree of variance in optical intensity and RNFL intensity has been shown to decrease with age, suggesting it as a poor choice for normalization.^82^ The ONL may be suggested as a possible candidate as it exhibits the least variance in optical intensity,^82^ however, the Henle fiber layer can increase the apparent ONL intensity (Fig. 3) and can be altered by disruption in cone structure.^75^ Additionally, the ELM may serve as a reliable layer for calculating relative EZ reflectivity, as it exhibits minimal intensity variation across eccentricity.^64^ Regardless, variability in normalization technique may preclude comparison of EZ reflectance across studies.

## Challenge 4: Relationship of the EZ to Photoreceptor Structure

Beyond the above issues surrounding image acquisition and analysis, perhaps the biggest hurdle impeding the clinical utility of EZ metrics (including reflectance) is their correlation with underlying photoreceptor structure. Cellular-resolution imaging of rod and cone structure is possible with the use of adaptive optics (AO) retinal imaging, which correct for the monochromatic aberrations of the eye.^83^^–^^85^ Such images enable extraction of information about photoreceptor density and topography in healthy and diseased retinae. In particular, AO scanning-light ophthalmoscopy (AOSLO) has been used to image photoreceptor structure in a wide range of retinal degenerative conditions.^83^^,^^86^

**Figure 5.**
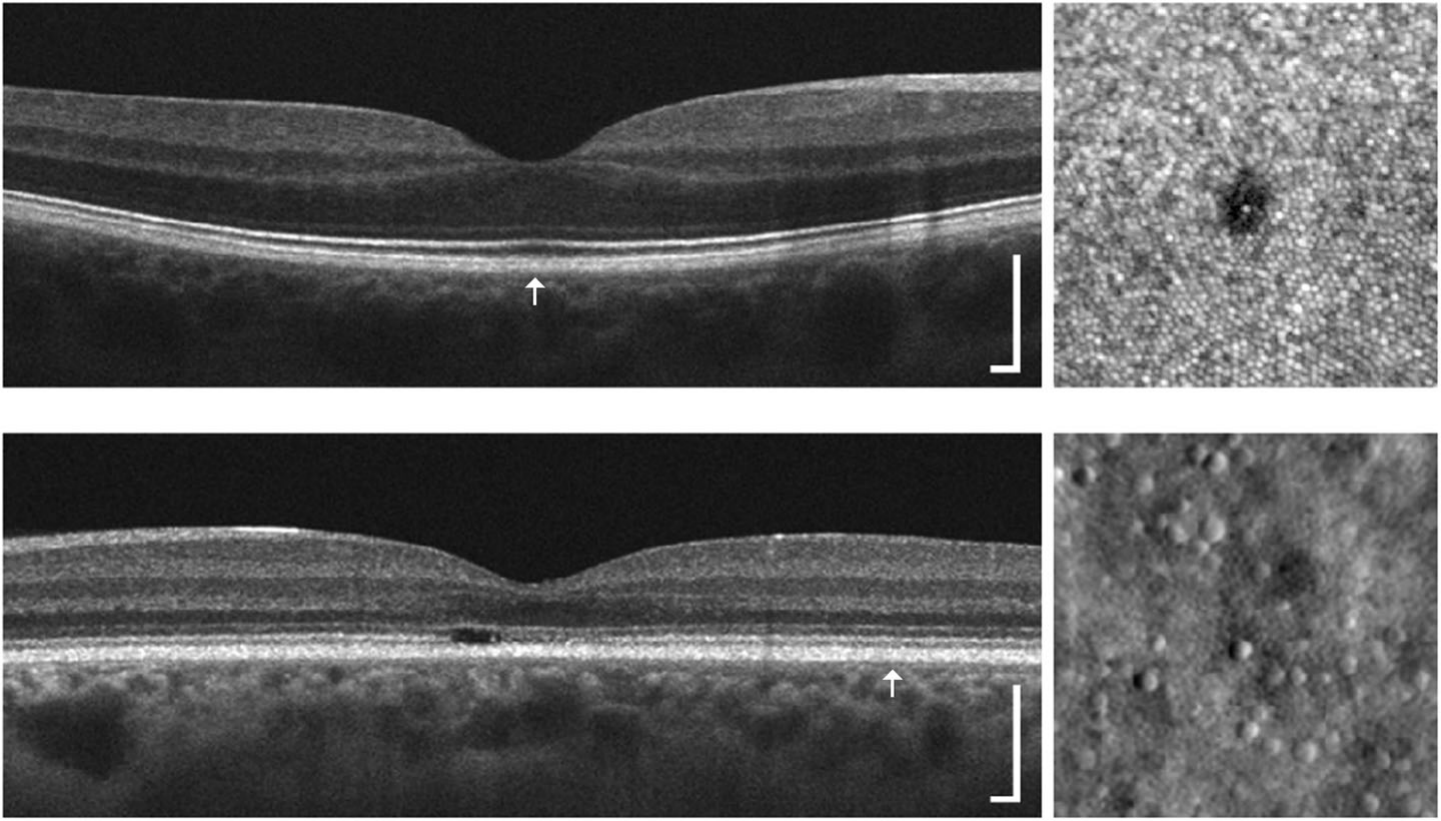
Comparison of EZ structure on OCT (*left*) with photoreceptor structure on AOSLO images (*right*). *Top:* This 31-year-old female was involved in a motor vehicle collision while traveling on her bike, which resulted in a head injury. The subject's chief complaint was unequal vision, difficulty maintaining clear vision, and photophobia. Clinical OCT imaging (HD-line scan acquired on the Cirrus) did not reveal any disruptions to the outer retina in the right eye. However, when imaged with confocal AOSLO, a small lamellar defect within the foveal mosaic was observed (similar to previous trauma cases).^97^ This defect was not observed with clinical OCT, likely due to the limited lateral resolution and sampling frequency of the retina with most clinical protocols. *Bottom:* This 43-year-old female had a family history of progressive vision loss and complained of decreased visual acuity and abnormal color vision. Genetic testing revealed a mutation in the *GUCY2D* gene (p.R838H; c.251G > A),^95^ which has been linked to autosomal dominant cone-rod dystrophy. Clinical OCT imaging (HD-line scan acquired on the Cirrus) revealed a focal EZ disruption within the macula of the left eye, while EZ integrity and reflectivity appeared relatively normal beyond the macular region (though there was thinning of the ONL at this location). When a region of the retina was visualized with nonconfocal split-detection AOSLO at ∼5.5° temporal to fixation, it was observed that cone density was significantly reduced (5500 cones/mm^2^ compared to 12,000 cones/mm^2^ for normal retinae^131^). Location of the AOSLO images for each subject is indicated by the *arrow* in the OCT scan, and they were acquired using previously described protocols.^84^^,^^95^^–^^97^ OCT *scale bar* = 200 µm; AOSLO region of interests are 150 × 150 µm.

Numerous studies have compared EZ structure on OCT with photoreceptor metrics from AO imaging. Many studies relate photoreceptor metrics from OCT, such as EZ reflectivity, with AO-derived metrics, finding good concordance between modalities in patients with maculopathies,^1^^,^^87^ RP,^57^ acute macular neuroretinopathy,^88^ macular hole,^89^ and central serous chorioretinopathy.^90^ However, there are some important examples of disconnects, including studies in patients with Usher syndrome,^41^ Stargardt disease,^15^ and MacTel Type 2^91^ that revealed an intact EZ on OCT even in areas where cone number was reduced and/or cones were damaged in corresponding AO images (Fig. 5). Moreover, some AO imaging studies have shown that loss of EZ integrity may not necessarily indicate an absence of underlying cone structure.^92^^–^^94^ For example, studies utilizing split detector AOSLO suggest the presence of remnant inner segment structure within foveal EZ lesions not visible with standard ophthalmic imaging in conditions such as MacTel Type 2,^91^ macular hole,^89^ cone-rod dystrophy,^93^ Best vitelliform macular dystrophy,^95^ and achromatopsia.^92^^,^^96^ Likewise, the presence of the EZ is not necessarily indicative of completely normal cone structure. For example, in some patients with ocular trauma, distinct cone loss is observed in areas with an intact and normally reflective EZ (though with an altered IZ band).^97^ In patients with Bornholm eye disease, there can be pronounced disruption in cone waveguiding despite completely normal EZ structure on OCT.^98^^,^^99^ Furthermore, subjects with albinism and dramatically reduced foveal cone density do not show overt attenuation or reduction of EZ reflectivity.^100^ Newer methods of quantifying EZ reflectivity may be worth examining in cases with specific amounts of cone and/or rod photoreceptor degeneration on AOSLO.^68^

These disconnects between underlying photoreceptor and EZ metrics suggest there may be important limitations on the sensitivity of EZ measures for quantifying photoreceptor degeneration across patients or over time within individual patients. Furthermore, it is important to note that existing EZ metrics (including reflectivity measures) from clinical OCT images cannot disambiguate the relative contribution of rods versus cones to the EZ band. These limitations may be overcome with future studies utilizing AO-OCT, which has demonstrated the ability to resolve separate bands associated with the rod and cone outer segments.^6^^,^^101^^,^^102^ Additional studies using clinical OCT, split detector and confocal AOSLO (for precise quantification of remnant rod and cone structure), and AO-OCT in populations with variable levels of photoreceptor degeneration could be key to elucidating the limits of EZ metrics extracted from clinical OCT imagery.

## Implications for Clinical Practice and Research

The challenges reviewed above suggest that standardized methods to evaluate EZ reflectivity are needed to facilitate its adoption as a biomarker of photoreceptor structure. Image processing and acquisition techniques intrinsic to different OCT machines have been shown to affect retinal thickness measurements, and there have been efforts to produce conversion equations to translate measurements across different machines.^103^ In line with this, a systematic approach involving conversion factors for lateral and axial scaling like that provided by Folgar et al. may be especially useful.^43^ In addition, many studies have investigated the reproducibility and repeatability of EZ measurements, indicating that further validation of these data is needed to accurately compare between devices. Logarithmic data limits the meaningfulness of reflectivity measurement because image compression narrows the range of comparison between values. Comparison of data obtained with different OCT devices may not be reliable, but normalization to a retinal layer that demonstrates minimal variability presents a potential way to solve this problem. The specifics of the device wavelength, software version, image depth, and image processing should be revealed prior to extracting EZ reflectivity measures, as this can facilitate comparison of data across studies. Such information may become more accessible within the OCT space as standardization of image file format occurs.^104^

The following are points to consider in changing practice. First, clinicians should be mindful of the post–image processing that occurs “behind the scenes” to produce an image that is ultimately displayed. An image that has pronounced contrast enhancement may be the result of logarithmic transform or other proprietary algorithms with unknown specification, and thus requires mathematical conversion back to raw data to accurately interpret reflectivity measurements. Second, future studies elucidating the relationship between pupil entry point and EZ appearance in a wide range of retinal degenerative diseases are warranted. Third, variations in spectrometer depth (due to EDI settings or variable working distance/reference arm settings) are generally overlooked yet can dramatically impact layer appearance on OCT. While some devices allow documentation of the reference arm settings, most do not, and this represents an area for improvement if EZ reflectivity metrics are to gain widespread clinical adoption.

It is critical to establish a standardized practice for measuring retinal layer reflectance, particularly for the evaluation of photoreceptor biomarkers. Various EZ metrics serve different purposes, but many rely on the use of the retinal reflectance profile. For example, segmentation software programs often use LRPs to delineate the individual layers of the retina.^105^ Similarly, there are metrics derived from the EZ band using LRPs, such as measuring outer segment length, that are used as biomarkers of photoreceptor density and spacing.^106^^,^^107^ The clinical utility of these metrics requires automation for the processing and analysis involved, like that seen with commercially available segmentation software, or databases available on commercial OCT devices that are used to assess retinal thinning.^108^ This is an area of rapid expansion, with new machine-learning and artificial-intelligence based algorithms emerging on an almost daily basis. Many studies have advanced automated methods for classifying various EZ metrics,^32^^,^^69^^,^^70^^,^^109^^–^^115^ though determining the extent to which these approaches accurately represent underlying photoreceptor structure (assessed with AO imagery) will be central to defining their clinical value.

A final point to consider is that static assessment of EZ reflectance only relates to structure while dynamic measures of reflectance may inform photoreceptor function. Emerging functional imaging techniques (e.g., dubbed intrinsic optical signal imaging, optophysiology, or optoretinography) capture structural changes in the photoreceptor in response to light.^116^^,^^117^ These manifest as changes in the appearance of the EZ and other outer retinal bands in AO-OCT images,^118^^,^^119^ or changes in photoreceptor reflectance in AOSLO images.^120^^,^^121^ This technique provides the opportunity to better understand biophysical changes to the retinal related to phototransduction,^116^^,^^122^^,^^123^ to classify specific photoreceptor classes,^118^^,^^124^^–^^126^ and to assess photoreceptor physiology in targeted regions of healthy and diseased retinae.^127^^–^^129^ While work is needed to understand how these functional imaging techniques relate to standard structural measures from clinical OCT imaging, optoretinography seems certain to become a valuable tool for improving the diagnosis, management, and treatment of retinal disease.
